# Rhinocerebral Mucormycosis in a Patient with Acute Promyelocytic Leukemia

**DOI:** 10.4274/tjh.2014.0048

**Published:** 2015-02-15

**Authors:** Muzaffer Keklik, Afra Yıldırım, Fahir Öztürk, İbrahim İleri, Gülşah Akyol, Mustafa Çetin, Bülent Eser

**Affiliations:** 1 Erciyes University Faculty of Medicine, Department of Hematology, Kayseri, Turkey; 2 Erciyes University Faculty of Medicine, Department of Radiology , Kayseri, Turkey; 3 Erciyes University Faculty of Medicine, Department of Internal Medicine, Kayseri, Turkey

**Keywords:** Acute promyelocytic leukemia, Rhinocerebral mucormycosis

A 63-year-old male presented with headache and weakness lasting for 2 months. Laboratory studies revealed the following: hemoglobin of 7.1 g/dL, white blood cells of 105x109/L, and platelets of 34x109/L. A diagnosis of acute promyelocytic leukemia was made by peripheral blood smear and bone marrow evaluations including morphological and genetic studies. The patient received one cycle of a chemotherapy regimen including cytarabine and idarubicin. During the aplastic phase, on day +6 from the end of chemotherapy, the patient developed fever, swelling on the left side of the face, infraorbital edema, and yellow-black discoloration of the upper palate (Figure 1). A paranasal sinus CT scan revealed a defect of the nasal septum and skin, and subcutaneous edema was seen at the maxillary and nasal level (Figure 2). The patient underwent surgical debridement. Histopathological assessment of the debridement specimen showed mucormycosis-associated hyphae. Culture of the nasal discharge was positive for Mucor spp. Liposomal amphotericin B was initiated at 5 mg/kg/day, but the patient died on the 30th hospital day. Informed consent was obtained.

Mucormycosis is rapidly progressive and mortality for this infection is high [1,2,3,4]. Hematologic malignancies, long-term corticosteroid use, and immunosuppressive therapies are predisposing factors for mucormycosis. For management, mucormycosis should be considered early in high-risk patients, and surgical debridement together with effective antifungal therapy should be applied as soon as possible.

## Figures and Tables

**Figure 1:**
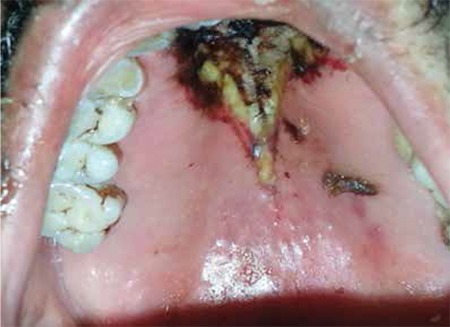
Yellow-black discoloration of upper palate.

**Figure 2:**
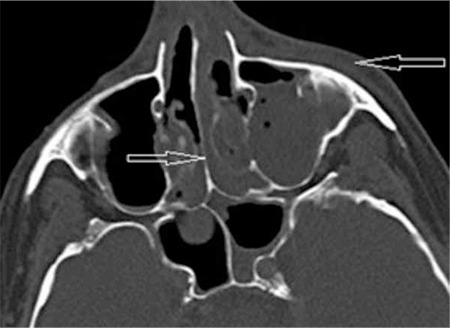
Paranasal sinus CT scan revealed a defect of the nasal septum and skin and subcutaneous edema.
